# Bleeding complications during cardiac electronic device implantation in patients receiving antithrombotic therapy: is there any value of local tranexamic acid?

**DOI:** 10.1186/s12872-016-0251-1

**Published:** 2016-04-22

**Authors:** Osman Beton, Ersin Saricam, Hakki Kaya, Hasan Yucel, Orhan Dogdu, Okan Onur Turgut, Ocal Berkan, Izzet Tandogan, Mehmet Birhan Yilmaz

**Affiliations:** Department of Cardiology, Heart Center, University Hospital, Faculty of Medicine, Cumhuriyet University, Postal Code: 58140 Sivas, Turkey; Cardiology Clinic, Cag Hospital, Ankara, Turkey; Cardiology Clinic, Koru International Hospital, Ankara, Turkey; Department of Cardiology, University Hospital, Faculty of Medicine, Firat University, Elazig, Turkey; Department of Cardiovascular Surgery, Heart Center, University Hospital, Faculty of Medicine, Cumhuriyet University, Sivas, Turkey; Cardiology Clinic, Gozde Academy Hospital, Malatya, Turkey

**Keywords:** Cardiac electronic device implantation, Topical tranexamic acid, Antithrombotic therapy, Pocket hematoma, Major bleeding complications

## Abstract

**Background:**

The perioperative use of antithrombotic therapy is associated with increased bleeding risk after cardiac implantable electronic device (CIED) implantation. Topical application of tranexamic acid (TXA) is effective in reducing bleeding complications after various surgical operations. However, there is no information regarding local TXA application during CIED procedures. The purpose of our study was to evaluate bleeding complications rates during CIED implantation with and without topical TXA use in patients receiving antithrombotic treatment.

**Methods:**

We conducted a retrospective analysis of consecutive patients undergoing CIED implantation while receiving warfarin or dual antiplatelet (DAPT) or warfarin plus DAPT treatment. Study population was classified in two groups according to presence or absence of topical TXA use during CIED implantation. Pocket hematoma (PH), major bleeding complications (MBC) and thromboembolic events occuring within 90 days were compared.

**Results:**

A total of 135 consecutive patients were identified and included in the analysis. The mean age was 60 ± 11 years old. Topical TXA application during implantation was reported in 52 patients (TXA group). The remaining 83 patients were assigned to the control group. PH occurred in 7.7 % patients in the TXA group and 26.5 % patients in the control group (*P* = 0.013). The MBC was reported in 5.8 % patients in the TXA and 20.5 % patients in control group (*P* = 0.024). Univariate logistic regression analysis identified age, history of recent stent implantation, periprocedural spironolactone use, periprocedural warfarin use, perioperative warfarin plus DAPT use, cardiac resynchronization therapy, and topical TXA application during CIED implantation as predicting factors of PH. Multivariate analysis showed that perioperative warfarin plus DAPT use (OR = 10.874, 95 % CI: 2.496–47.365, *P* = 0.001) and topical TXA application during CIED procedure (OR = 0.059, 95 % CI: 0.012–0.300, *P* = 0.001) were independent predictors of PH. Perioperative warfarin plus DAPT use and topical TXA application were also found to be independent predictors of MBC in multivariate analyses. No thromboembolic complications was recorded in the study group.

**Conclusion:**

The present study demonstrated that the topical TXA application during CIED implantation is associated with reduced PH and MBC in patients with high bleeding risk.

**Electronic supplementary material:**

The online version of this article (doi:10.1186/s12872-016-0251-1) contains supplementary material, which is available to authorized users.

## Background

The number of cardiac implantable electronic device (CIED) implantation has increased considerably in the last decade [1] and has been continuing to increase parallel to expanded indications in the most recent guidelines [2, 3]. As a result, numerous patients have undergone CIED implantation while receiving antithrombotic therapy for few years [4]. Perioperative management of these patients is very challenging with a high risk of bleeding [5]. Pocket hematoma is relatively common complication (reported rates between 2 % and 5 %) [6–8], and dual antiplatelet therapy (DAPT) increases the risk fivefold [6], and furthermore rate of bleeding complication can be as high as 40 % with triple therapy [9].

In the presence of moderate or high thromboembolic risk, physicians usually accept the risk of bleeding and continue the antithrombotic therapy perioperatively. It is clearly known that postoperative bleeding complications constitutes not only financial but also significant medical burden. Several experience based strategies are available to reduce the incidence of pocket hematoma: meticulous use of cautery, tamponade of pocket with gauze during lead implantation, wound drainage, pressure bandage and hemostatic use. Also, some hemostatic agents were found to be ineffective in preventing pocket hematoma, besides increasing the risk of pocket infection [10]. Also, these agents are not available in every clinic, need time to be prepared and all increases the cost.

Tranexamic acid (TXA) is a synthetic antifibrinolytic agent that binds to the lysine binding site of plasminogen and blocks its binding to fibrin surface [11]. Topical application of TXA is effective in reducing bleeding complications after various surgical operations [11]. However, there is no information regarding local TXA application during CIED procedures. The purpose of our study was to evaluate bleeding complications rates during CIED implantation with and without topical TXA use in patients receiving antithrombotic treatment.

## Methods

### Study cohort

We conducted a retrospective analysis of consecutive patients undergoing CIED implantation while receiving antithrombotic therapy, namely, warfarin or warfarin plus DAPT or DAPT. Between June 2011 and February 2015, 989 consecutive patients who underwent CIED procedures at Cumhuriyet University Hospital, Sivas, Turkey and Cag Hospital, Ankara, Turkey were considered. At index procedure, a total of 319 patients were on chronic background therapy with warfarin or warfarin plus DAPT or DAPT. Indication of DAPT consisting of combination of acetylsalicylic acid and clopidogrel was recent stent implantation (within 3–12 months) in all patients receiving DAPT. Exclusion criteria were: use of other antiplatelets (e.g. prasugrel, ticagrelor, ticlopidine), heparin bridging strategy, interrupted antiplatelet therapy more than 2 days before the procedure, unknown international normalization ratio (INR) or INR values inconsistent with target range at the day of procedure, periprocedural bleeding not related to CIED procedure (eg, gastrointestinal bleeding, hematuria, etc.), lead extraction with laser or mechanical dilator (As a protocol, all antithrombotic drugs have been discontinued at least 7 days prior to this procedure in both clinics). Inclusion criteria were one of the below strategies for perioperative antithrombotic management:

- *Uninterrupted warfarin;* Warfarin therapy was continued to maintain INR in the therapeutic range (2.0–3.5), but dosage of warfarin was adjusted to maintain INR between 1.9 and 2.5 at the day of procedure.
- *Uninterrupted warfarin plus DAPT;* In addition to uninterrupted warfarin strategy, aspirin and/or clopidogrel was continued perioperatively without dose skipping more than 2 days before the procedure.
- *Uninterrupted DAPT;* Aspirin and/or clopidogrel was continued perioperatively without dose skipping more than 2 days before the procedure.

**Fig. 1:**
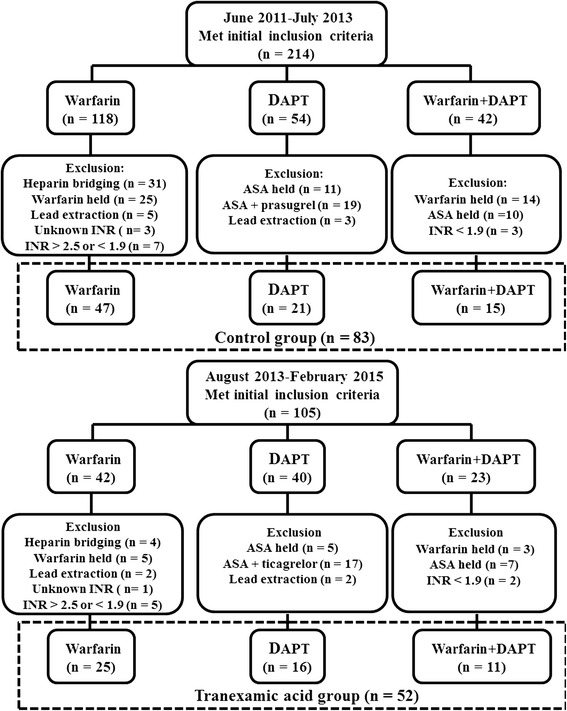
Flow chart of the patients who participated in the study as postive control (*top*) or tranexamic acid (*bottom*) group and the assignment of the patients to medication subgroups on the basis of medications taken during the periprocedure period is displayed. INR values at the day of procedure were used in exclusion boxes. DAPT = dual antiplatelet treatment

Hospital records were reviewed to determine medications at admission and during perioperative period. Patients were grouped according to antithrombotic medications at admission (Fig. 1, top and bottom). A protocol for topical utilization of TXA during CIED implantations was proposed for the high risk patients at Cumhuriyet University by August 2013 up on several discussions of Heart Team and advised to Cag Hospital as well and the protocol has been routinely used in both hospital since August 2013. Hence, TXA group consisted of patients who underwent CIED procedure after August 2013 (between August 2013 and February 2015) and the control group consisted of patients who underwent CIED procedure before August 2013 (between June 2011- July 2013). The study was approved by the Ethics Committee of the Faculty of Medicine, Firat University (reference number: 201602/06) and conducted in accordance with the Helsinki Declaration.

### Definitions

Procedure related bleeding complications was recorded as pocket hematoma and major bleeding complications (MBC). Pocket hematoma (PH) was defined as swelling and a painful mass with ecchymosis formation extending the margin of generators.

MBC defined as any CIED procedure related bleeding leading to red blood cell (RBC) transfusion, surgical intervention for pocket evacuation or revision, pericardial effusion, hemothorax, or life-threatening bleed [6]. If a patient experienced multiple MBC, the clinical time course was reviewed to ensure that complications counted were distinctly separate events related to the CIED procedure.

Thromboembolic events were defined as transient ischemic attack, stroke, myocardial infarction, systemic embolism, mechanical valve thrombosis, deep vein thrombosis, and pulmonary embolism.

### Implantation techniques

All procedures were performed by experienced cardiologists, blinded to study plan. All of the implanting physicians had an experience of at least 150 operations per annum with a similar experience in years (>3 years). All the patients were administered intravenous prophylactic antibiotics (mostly cefazolin, a first generation cephalosporin) per hospital protocol. After administration of local anesthesia (lidocaine or prilocaine hydrochloride), an incision was made in the prepectoral region and a subcutaneous or submuscular pocket was formed according to device and subcutaneous tissue features of the patient. Electro cautery ± ligatures were routinely used to obtain complete surgical hemostasis. All the leads were implanted via sublavian/axillary venous puncture (one puncture per lead) or cephalic cut-down. Right ventricular leads were usually targeted to the interventricular septum of right ventricle. The left ventricular leads were inserted through the coronary sinus. After lead measurements with analyzer, leads were sutured using nonabsorbable silk sutures at the venous site. Generators were placed in preformed pocket and were sutured using nonabsorbable silk sutures. The incision was closed in three layers using absorbable sutures, and a sterile pressure dressing was applied. Implantation technique were same for all patients except additional local TXA application in TXA group.

#### Utilization of topical transexamic acid during implantation

According to our protocol, after pocket formation and complete surgical hemostasis with electrocoutery ± surgical ligation, pocket and the incision sites were washed with 500 mg/5 ml TXA without removing with suction. Then, a standard gauze soaked in 5 ml solution containing 500 mg of TXA was placed in the prepared pocket, and steady and direct pressure by using the palm of one hand was applied on the gauze from the skin for 1–3 min. The gauze was left in the pocket throughout the procedure and was removed from the pocket before the device placement in the pocket. Lastly, pocket and incision sites were washed with 500 mg/5 ml TXA just before incision closure without removing with suction.

All the cardiologists performing CIED implantation have known the topical TXA protocol well (experience of at least 15 implantations with TXA). All details about the procedure was routinely documented by the attending cardiologist and it was recorded both in computer based system and patient’s CIED file.

### Postoperative care and data collection

A pressure dressing was applied to the wound for 24–36 h. A chest X-ray was obtained after the procedure. Intravenous antibiotics and oral or iv pain medications (mostly asetaminofen) were given for 1–3 days. All cardiac medications were continued. Patients were discharged at third day after the procedure. All patients were evaluated at discharge and 7, 15, 30 and 90 days after the procedure to access wound and suture integrity, pocket hematoma, thromboembolic events, CIED functions, device infection, or other problems in outpatient outpatient cardiology clinic and findings were recorded in patient’s CIED file.

Data were retrospectively collected from patient’s file, medical observations and computer based database system. In addition to usual patient characteristics, the following information were collected: all medications, technical surgical details and laboratory data.

### Statistical analysis

Continuous variables were expressed as mean ± standard deviation or median (min-max) in the presence of abnormal distribution, and categorical variables as percentages. Comparisons between groups of patients were made by use of a χ2 test for categorical variables, independent samples *t* test for normally distributed continuous variables, and Mann-Whitney *U* test when the distribution was skewed. We used univariate logistic regression analysis to quantify the association of variables with occurrence major bleeding and PH. Age, presence of LV thrombus, history of recent stent implantation, spironolactone use, periprocedural warfarin use, periprocedural warfarin plus DAPT use, ICD device implantation, three lead implantation, and topical TXA use during CIED implantation were entered into the multivariate logistic regression model for determining the independent predictors of PH. Hypertension, history of recent stent implantation, spironolactone use, periprocedural warfarin use, periprocedural warfarin plus DAPT use, and topical TXA use during implantation were entered into the multivariate logistic regression model in order to determine the independent predictors of MBC. All statistical procedures were performed using SPSS software version 14.0 (SPSS Inc., Chicago, IL). A *p* value of 0.05 was considered as statistically significant.

## Results

### Study population

A total of 135 consecutive patients were identified and included in the present analysis. Topical TXA use was noted in 52 patients during implantation the remaining 83 patients were assigned to be as control group that included traditional routine precautions in these high risk patients. The mean age was 60 ± 11 years with 81 (60 %) males. Baseline characteristics of the study population are presented in Table 1 and all variables were statistically similar between the groups. Procedure related characteristics of the study population are presented in Table 2. There was no difference between the groups according to these variables.

**Table 1:** Baseline characteristics of study population

Variables	All patients (*n* = 135)	Tranexamic acid (*n* = 52)	Control (*n* = 83)	*P*-value
Age (years)	60 ± 11	62 ± 10	59 ± 11	0.101
Male, *n* (%)	81 (60)	32 (61.5)	46 (59)	0.773
Body mass index (kg/m^2^)	25.4 ± 4.6	24.8 ± 4.0	25.8 ± 5.0	0.215
Smoking, *n* (%)	22 (16.3)	9 (17.3)	13 (15.7)	0.990
Hypertension, *n* (%)	74 (54.8)	29 (55.8)	45 (54.2)	0.860
Diabetes mellitus, *n* (%)	46 (34.1)	17 (32.7)	29 (34.9)	0.789
Previous CABG, *n* (%)	43 (31.9)	18 (34.6)	25 (30.1)	0.585
Ejection fraction, %	34 (20–55)	34 (20–55)	34 (20–55)	0.910
COPD, *n* (%)	19 (14.1)	9 (17.3)	10 (12.0)	0.548
Hemoglobin, g/dL	12.8 (9.5–15.1)	12.9 (9.5–14.5)	12.8 (9.5–15.1)	0.989
Platelet count, K/mm^3^	244 ± 57	240 ± 56	247 ± 58	0.476
BUN, mg/dL	30.6 (22–45)	30.6 (25–43)	30.6 (22–45)	0.926
Creatinine, mg/dL	1.1 ± 0.4	1.0 ± 0.4	1.1 ± 0.4	0.447
Atrial fibrillation, n (%)	43 (31.9)	22 (42.3)	21 (25.3)	0.039
Metallic prosthetic valve, *n* (%)	54 (40.0)	20 (38.5)	34 (41.0)	0.773
LV thrombus, *n* (%)	9 (6.7)	6 (11.5)	3 (3.6)	0.087
Recent stent implantation, *n* (%)	63 (46.7)	27 (51.9)	36 (43.4)	0.333
Medications				
ACEI/ARB, *n* (%)	95 (70.4)	36 (69.2)	59 (71.1)	0.818
Beta blocker, *n* (%)	112 (83)	43 (82.7)	69 (83.1)	1.0
Diuretic, *n* (%)	99 (73.3)	37 (71.2)	62 (74.7)	0.800
Spironolactone, *n* (%)	67 (49.6)	25 (48.1)	42 (50.6)	0.775
Statin, *n* (%)	62 (45.9)	24 (46.2)	38 (45.8)	0.966
Warfarin, *n* (%)	72 (53.3)	25 (48.1)	47 (56.6)	0,333
DAPT, *n* (%)	37 (27.4)	16 (30.8)	21 (25.3)	0.621
Warfarin plus DAPT, *n* (%)	26 (19.3)	11 (21.2)	15 (18.3)	0.828

*ACEI* angiotensin converting enzyme inhibitors, *ARB* angiotensin receptor blockers, *BUN* blood urea nitrogen, *CABG* coronary artery bypass graft, *COPD* chronic obstructive pulmonary disease, *DAPT* dual antiplatelet therapy, *LV* left ventricular

**Table 2:** Procedure related characteristics between the tranexamic acid and control groups

Characteristic	All patients (*n* = 135)	Tranexamic acid (*n* = 52)	Control (*n* = 83)	*P*-value
INR at the day of implant^a^	2.1 (1.9–2.5)	2.1(1.9–2.5)	2.1(1.9–2.5)	0.932
Generator exchange and/or pocket revision, *n* (%)	14 (10.4)	5 (9.6)	9 (10.8)	0.968
New implantation, *n* (%)	101 (74.8)	39 (75.0)	62 (74.7)	1.0
Upgrade and/or lead revision, *n* (%)	20 (14.8)	8 (15.4)	12 (14.5)	1.0
Pacemaker, *n* (%)	24 (17.8)	9 (17.3)	15 (18.1)	1.0
ICD, *n* (%)	111 (83)	43 (82.7)	68 (81.9)	
Number of leads implanted				
One, *n* (%)	26 (19.3)	12 (23.1)	14 (16.9)	0.505
Two, *n* (%)	50 (37.0)	18 (34.6)	32 (38.6)	0.645
Three, *n* (%)	45 (33.3)	17 (32.7)	28 (33.7)	0.900
Submuscular pocket, *n* (%)	5 (3.7)	2 (3.8)	3 (3.6)	1.0
Venous route other than subclavian				
Axillary, *n* (%)	12 (8.9)	4 (7.7)	8 (9.6)	0.767
Cephalic, *n* (%)	7 (5.2)	3 (5.8)	4 (4.8)	1.0

*DAPT* dual antiplatelet therapy, *ICD* implantable cardioverter defibrillator, *INR* international normalized ratio

^a^The median INR level of patients with warfarin continuation strategy

### Procedure related complications

Procedure related complications are presented in Table 3. A total of 28 MBC events (reoperation, RBC transfusion, hemothorax, pericardial effusion, and life-threatening bleed) were reported in 20 patients (14.8 %) in the study population (Table 3). MBC occurred in 3 patients (5.8 %) in the TXA group and in 17 patients (20.5 %) in the control group (*P* = 0.024) (Table 3). Total counted event rates of reoperation and RBC transfusion were statistically higher in control group (9.6 % vs 0.0 %, *P* = 0.023 and 15.7 % vs 3.8 %, *P* = 0.047, respectively). PH occurred in 4 patients (7.7 %) in the TXA group and 22 patients (26.5 %) in the control group (*P* = 0.013). The detailed characteristics of patients with pocket hematoma and MBC are summarized in Additional file 1: Table S1.

**Table 3:** Procedure related complications

Complications	All patients (*n* = 135)	Tranexamic acid (*n* = 52)	Control (*n* = 83)	*P*-value
Major bleeding complications, *n* (%)	20 (14.8)	3 (5.8)	17 (20.5)	0.024
Reoperation^a^, *n* (%)	8 (5.9)	0 (0.0)	8 (9.6)	0.023
RBC transfusion^a^, *n* (%)	15 (11.1)	2 (3.8)	13 (15.7)	0.047
Hemothorax^a^, *n* (%)	1 (0.7)	0 (0.0)	1 (1.2)	1.0
Pericardial effusion^a^, *n* (%)	3 (2.2)	1 (1.9)	2 (2.4)	1.0
Life-threatening bleed^a^, *n* (%)	1 (0.7)	0 (0.0)	1 (1.2)	1.0
Pocket hematoma, *n* (%)	26 (19.3)	4 (7.7)	22 (26.5)	0.013
Pocket related infection, *n* (%)	1 (0.7)	0 (0.0)	1 (1.2)	1.0
Pneumothorax, *n* (%)	2 (1.4)	1 (1.9)	1 (1.2)	1.0

*RBC* red blood cells. ^a^Counted events were presented (If a patient experienced multiple major bleeding complications, the clinical time course was reviewed to ensure that complications counted were distinctly separate events related to the procedure)

One pocket related infections (after hematoma evacuation procedure) requiring full system extraction was reported in control group. Pneumothorax was occurred in 2 patients (one in control group and 1 in TXA group, and chest tube drainage was required for both). One patient (78 years old male patient receiving periprocedural DAPT) died after implantation because of complicating hemathorax and large pericardial effusion in the control group. Two patients had pericardial effusion which required pericardiosynthesis. No thromboembolic complications were occurred in the study group perioperatively and 90 days after the procedure.

### Predictors of pocket hematoma

Baseline characteristics of the study population according to presence and absence of PH are presented in Table 4. Mean age was statistically higher in PH positive group (*n* = 26) compared to PH negative group (*n* = 109) (64 ± 12 vs 59 ± 11, *P* = 0.037). More patients had history of recent stent implantation in PH positive group (88.5 % vs 36.7 %, *P* < 0.001). Periprocedural warfarin plus DAPT and spironolactone use were higher in PH positive group compared to PH negative (57.7 % vs 10.1 %, *P* < 0.001 and 73.1 % vs 44.0 %, *P* = 0.015, respectively). Periprocedural warfarin use was higher in PH negative group (62.4 % vs 15.4 %, *P* < 0.001) (Table 4).

**Table 4:** Baseline characteristics according to pocket hematoma

Variables	PH positive group (*n* = 26)	PH negative group (*n* = 109)	*P*-value
Age	64 ± 12	59 ± 11	0.037
Male, *n* (%)	14 (53.8)	67 (61.5)	0.624
Body mass index (kg/m^2^)	26.1 ± 5.7	25.3 ± 4.3	0.396
Smoking, *n* (%)	3 (11.5)	19 (17.4)	0.567
Hypertension, *n* (%)	18 (69.2)	56 (51.4)	0.154
Diabetes mellitus, *n* (%)	7 (26.9)	39 (35.8)	0.531
Previous CABG, *n* (%)	9 (34.6)	34 (31.2)	0.918
Ejection fraction, %	32 (25–55)	34 (20–55)	0.210
COPD, *n* (%)	3 (11.5)	16 (14.7)	1.0
Hemoglobin, g/dL	13.2 (10.8–15.1)	12.8 (9.5–14.5)	0.946
Platelet count, K/mm^3^	239 ± 69	246 ± 55	0.630
BUN, mg/dL	30.2 (25–39)	31.6 (22–45)	0.975
Creatinine, mg/dL	1.0 ± 0.4	1.1 ± 0.4	0.614
Atrial fibrillation, *n* (%)	10 (38.5)	33 (30.3)	0.568
Metallic prosthetic valve, *n* (%)	11 (42.3)	43 (39.4)	0.964
LV thrombus, *n* (%)	4 (15.4)	5 (4.6)	0.069
Recent stent implantation, *n* (%)	23 (88.5)	40 (36.7)	<0.001
Medications			
ACEI/ARB, *n* (%)	22 (84.6)	73 (67.0)	0.126
Beta blocker, *n* (%)	24 (92.3)	88 (80.7)	0.245
Diuretic, *n* (%)	21 (80.8)	78 (71.6)	0.479
Spironolactone, *n* (%)	19 (73.1)	48 (44.0)	0.015
Statin, *n* (%)	13 (50.0)	49 (45.0)	0.807
Warfarin, *n* (%)	4 (15.4)	68 (62.4)	<0.001
DAPT, *n* (%)	7 (26.9)	30 (27.5)	1.0
Warfarin plus DAPT, *n* (%)	15 (57.7)	11 (10.1)	<0.001

*ACEI* angiotensin converting enzyme inhibitors, *ARB* angiotensin receptor blockers, *BUN* blood urea nitrogen, *CABG* coronary artery bypass graft, *COPD* chronic obstructive pulmonary disease, *DAPT* dual antiplatelet therapy, *LV* left ventricular, *PH* pocket hematoma

Procedure related characteristics of the study population according to PH are presented in Table 5. More ICD devices were implanted in PH positive group compared to PH negative group (96.2 % vs 78.9 %, *P* = 0.045). The topical TXA use during CIED implantation was higher in MBC negative group compared to MBC positive group (44.0 % vs 15.4 %, *P* = 0.013).

**Table 5:** Procedure related characteristics according to pocket hematoma

Characteristics	PH positive group (*n* = 26)	PH negative group (*n* = 109)	*P*-value
INR at the day of implant^a^	2.1 (2.0–2.5)	2.0 (1.9–2.5)	0.122
Generator exchange and/or pocket revision, *n* (%)	3 (11.5)	11 (10.1)	1.0
New implantation, *n* (%)	19 (73.1)	82 (75.2)	1.0
Upgrade and/or lead revision, *n* (%)	4 (15.4)	16 (14.7)	1.0
Pacemaker, *n* (%)	1 (3.8)	23 (21.1)	0.045
ICD, *n* (%)	25 (96.2)	86 (78.9)	
Number of leads implanted			
One, *n* (%)	2 (7.7)	24 (22.0)	0.165
Two, *n* (%)	8 (30.8)	42 (38.5)	0.610
Three, *n* (%)	13 (50.0)	32 (29.4)	0.076
Submuscular pocket, *n* (%)	1 (3.8)	4 (3.7)	1.0
Venous route other than subclavian			
Axillary, *n* (%)	3 (11.5)	9 (8.3)	0.700
Cephalic, *n* (%)	2 (7.7)	5 (4.6)	0.620
Local tranexamic acid use	4 (15.4)	48 (44.0)	0.013

*DAPT* dual antiplatelet therapy, *ICD* implantable cardioverter defibrillator, *INR* international normalized ratio, *PH* Pocket hematoma. ^a^ The median INR level of patients with warfarin continuation strategy

Results of univariate and multivariate analyses for prediction of PH occurrence are presented in Table 6. In the study group, univariate analyses identified seven predictors of PH: age (OR = 1.043, 95 % CI: 1.002–1.085, *P* = 0.040), history of recent stent implantation (OR = 13.225, 95 % CI: 3.734–46.839, *P* < 0.001), periprocedural spironolactone use (OR = 3.449, 95 % CI: 1.340–8.879, *P* = 0.010), periprocedural warfarin use (OR = 0.110, 95 % CI: 0.035–0.341, *P* < 0.001), perioperative warfarin plus DAPT use (OR = 12.149, 95 % CI: 4.483–32.920, *P* < 0.001), three lead implantation during procedure (OR = 2.406, 95 % CI: 1.006–5.757, *P* = 0.049), and topical TXA application during CIED implantation (OR 0.231, 95 % CI: 0.075–0.716, *P* = 0.011). Multivariate analysis of factors with *P* < 0.1 in univariate analysis showed that perioperative warfarin plus DAPT use (OR = 10.874, 95 % CI: 2.496–47.365, *P* = 0.001) and topical TXA application during CIED procedure (OR = 0.059, 95 % CI: 0.012–0.300, *P* = 0.001) were independent predictors of MBC.

**Table 6:** Univariate and multivariate predictors of pocket hematoma

	Univariate			Multivariate		
Variables	OR	95 % CI	*P*-value	OR	95 % CI	*P*-value
Age	1.043	1.002–1.085	0.040			
Presence of LV thrombus	3.782	0.939–15.228	0.061			
History of recent stent implantation	13.225	3.734–46.839	<0.001			
Spironolactone use	3.449	1.340–8.879	0.010			
Periprocedural warfarin use	0.110	0.035–0.341	<0.001			
Periprocedural warfarin plus DAPT use	12.149	4.483–32.920	<0.001	10.874	2.496–47.365	0.001
ICD device	6.686	0.860–51.991	0.069			
Three lead implantation	2.406	1.006–5.757	0.049			
Topical TXA use during CIED implantation	0.231	0.075–0.716	0.011	0.059	0.012–0.300	0.001

*CI* confidence interval, *CIED* cardiac electronic device implantation, *DAPT* dual antiplatelet therapy, *ICD* implantable cardioverter defibrillator, *LV* left ventricular, *MBC* major bleeding complications, *PH* pocket hematoma, *TXA* tranexamic acid

### Predictors of major bleeding complications

In the study group, there were 20 patients with MBC (MBC positive group) and 115 without MBC (MBC negative group). Baseline characteristics of the study population according to major bleeding complications are presented in Additional file 1: Table S2. More patients had history of recent stent implantation in MBC positive group (80.0 % vs 40.9 %, *P* = 0.003). Periprocedural warfarin plus DAPT use was higher in MBC positive group compared to MBC negative (50.0 % vs 13.9 %, *P* = 0.001). Periprocedural warfarin use was higher in MBC negative group (59.1 % vs 20.0 %, *P* = 0.003) (Additional file 1: Table S2).

Procedure related characteristics of the study population according to MBC are presented in Additional file 1: Table S3. The topical TXA use during CIED implantation was higher in MBC negative group compared to MBC positive group (42.6 % vs 15.0 %, *P* = 0.036).

Results of univariate and multivariate analyses for prediction of MBC occurrence are presented in Additional file 1: Table S4. In the study group, univariate analyses identified four predictors of MBC: history of recent stent implantation (OR = 5.787, 95 % CI: 1.820–18.406, *P* = 0.003), periprocedural warfarin use (OR = 0.173, 95 % CI: 0.054–0.550, *P* = 0.003), perioperative warfarin plus DAPT use (OR = 6.187, 95 % CI: 2.224–17.216, *P* < 0.001), and topical TXA application during CIED implantation (OR 0.238, 95 % CI: 0.066–0.856, *P* = 0.028). Multivariate analysis of factors with *P* < 0.1 in univariate analysis showed that perioperative warfarin plus DAPT use (OR = 8.144, 95 % CI: 2.589–25.618, *P* < 0.001) and topical TXA application during CIED procedure (OR = 0.170, 95 % CI: 0.042–0.690, *P* = 0.013) were independent predictors of MBC.

## Discussion

The present study retrospectively investigated the effect of additional topical TXA use during CIED procedure on PH and MBC in a population with uninterrupted antithrombotic therapy strategy. The overall rates of PH and MBC were 19.3 % and 14.8 %, respectively. The frequencies of PH and MBC were significantly higher (3.4-fold and 3.5-fold, respectively) in the control group compared to TXA group. The counted event rates of reoperation and RBC transfusion requirements were lower in TXA group. In multivariate analysis, warfarin plus DAPT was found to be an independent predictor of increased risk of PH and MBC, whereas, topical TXA application during CIED was found to be an independent predictor of decreased risk PH and MBC.

Expanding CIED implantation indications are mainly belonging to implantable cardioverter defibrillators (ICD) and mostly includes patients with ischemic heart disease [2, 3]. Some of those patients have recently undergone coronary stent implantation. Following coronary intervention the need for DAPT usually is temporally, the duration of therapy mainly depending on the type of the stent (drug eluting or bare) [5]. Shortening the initially recommended time period of DAPT is highly discouraged due to high risk of stent thrombosis [5]. Furthermore, some ICD implants should not be postponed because of a potential risk of sudden cardiac death. However, it is well known that bleeding complications occurs more frequently in patients receiving ICD than those receiving PMs. [12] In the present study, 83 % of the devices were ICD and 46.7 % of the patients had recent stent implantation.

BRUISE Control trial reported that the incidence of hematoma is 3.5 % in patients with perioperative strategy of uninterrupted warfarin group [13]. On the other hand, in the FinPAC trial, the risk of hematoma was similar in patients on aspirin (5.5 %) and warfarin (5.6 %), but 0.9 % in patients with no antithrombotic therapy [14]. The presence of wide variability among reported incidence of pocket hematoma and bleeding complications in the numerous published studies is according to different criterias used. But, bleeding complications which require serious interventions such as drainage (pocket, pericardial or pleural) or transfusion are the only practically important ones. So, both PH and MBC criterias were used for this study in coherent with expectations about practical issues.

In the present study, periprocedural antithrombotic treatment strategy was uninterrupted warfarin 53.3 % of the patients, uninterrupted DAPT in 27.4 % of the patients, and uninterrupted warfarin plus DAPT (triple therapy) in 19.3 % of the patients. Despite the high rates of periprocedural antiplatelet therapy with warfarin and ICD implantation, PH and MBC rates were relatively low in TXA group. These findings may be attributed to the topical TXA usage. According to a recent systemic review, there is reliable evidence that topical application of TXA reduces bleeding and blood transfusion in surgical patients [11]. Although bleeding from CIED surgical sites is usually controllable, there may be significant blood loss. The topical administration of TXA intraoperatively can directly target the source of bleeding and may stabilize the multiple micro-clots that form within the wound containing high capillary density [11, 15].

Following the surgical trauma, clotting cascade is activated to produce a fibrin-based clot at the location of vascular injury. Fibrinolysis begins soon after as a normal homeostatic response to restore vascular patency and to prevent positive feedback from causing clot activation throughout the vascular compartment [15, 16]. However, in the first hour after severe injury, hyperfibrinolysis can occur which can lead to further bleeding, and TXA acts to block this [15, 16]. Topical TXA may be more beneficial especially in the presence antithrombotic therapy, when capillary or venous ooze is more pronounced [17, 18]. In addition, TXA may have positive impact on platelet functions by inhibiting the conversion of plasminogen to plasmin which impairs platelet functions [18].

In the past decade, some hemostatic agents were studied in preventing pocket hematoma. A fibrin selant prior to wound closure was shown to be effective in reducing the number of pocket hematomas regardless of the anticoagulation strategy used, but it may be associated with an increased cost, viral transmission, allergic reactions to bovine proteins or infections [19]. Recently, topical application of a hemostat containing mixture of thrombin and collagen was studied in patients on anticoagulants and/or antiplatelets [10]. However, it was found that this hemostat does not decrease the frequency of clinically relevant pocket hematomas, but increase the rate of pocket infections [10]. In the present study, no allergic reaction was recorded. Also, no pocket infection was observed in topical TXA group during follow-up period. The low cost of the TXA was considered as an additional advantage in comparison with other hemostatic agents.

Similar to other studies, the present study found that history of recent stent implantation and triple therapy is associated with PH and MBC. Relationship of history of recent stent implantation with PH and MBC could be clearly attributed to DAPT usage in these pateints. Because, patients with recent stent implantation were on DAPT ± warfarin therapy in the present study population. In univariate analyses, presence of left ventricular (LV) thrombus was found to be related to increased PH risk. In the present study, all patients with LV thombus had previous history of anterior myocardial infraction and recent stent implantation, and thereby they had periprocedural uninterrupted warfarin plus DAPT strategy which was found to be independently related with PH complication. Additionally, spironolactone use and three lead implantation (which implies cardiac resynchronization therapy) during the procedure were found to be related with increased risk of PH in univariate analyses. More ICDs (especially cardiac resynchronization therapy-defibrillator [CRT-D]) were implanted in PH positive group, so the rate of spironolactone use was higher in PH positive group. Twenty (76.9 %) of the patients with PH had CRT-D implantation procedure (13 new implantation, 4 upgrade and/or lead revision, and 3 generator replacement) (Additional file 1: Table S1). This finding was consistent with other studies. In a recent review, it was stated that rates of PH is around 2.5 % in ICD implantations, >3 % in case of CRT-D or cardiac resynchronization therapy-pacemaker (CRT-P) implantation, up to 4.2 % in case of upgrading of a previous implant to CRT-P/CRT-D, and 4.3 % in case of surgical revision of the implant with repositioning due to dislocation or placement of a new lead [20].

To our knowledge, this is the first study to demonstrate that topical TXA use was associated with lower PH and MBC rates during CIED procedures of patients with uninterrupted antithrombotic therapy.

### Limitations

Several limitations of this study should be acknowledged. Firstly, it was a retrospective anaylsis which is susceptible to bias in data selection. Secondly, sample size of our study was relatively small, hence, no subgroup analysis was possible. Thirdly, the volumes of the conducting centers of the present study were not enough to draw definitive conclusions rather than hypothesis generation. Hence, large scaled randomized and prospective trials are needed to access safety, efficacy and cost-effectivity of topical TXA in CIED procedures.

## Conclusion

The present study demonstrated for the first time that addition of topical TXA during CIED implantation was associated with reduced PH and MBC in patients with high bleeding risk. However, further trials needed to test safety and efficacy of topical TXA application in CIED procedures.

## Availability of data and materials

The datasets supporting the conclusions of this article are included within the article and its additional supplementary files.

## Consent for publication

Not applicable.

## Additional files

Additional file 1: Table S1. Characteristics of patients with pocket hematoma and/or major bleeding complications. **Table S2**. Baseline characteristics according to MBC. **Table S3**. Procedure related characteristics according to MBC. **Table S4**. Univariate and multivariate predictors of MBC. (DOCX 28 kb)

## Abbreviations

CIED: cardiac implantable electronic device
CRT-D: cardiac resynchronization therapy-defibrillator
CRT-P: cardiac resynchronization therapy-pacemaker
DAPT: dual antiplatelet treatment
ICD: implantable cardioverter defibrillators
INR: international normalization ratio
LV: left ventricular
MBC: major bleeding complications
PH: pocket hematoma
RBC: red blood cell
TXA: tranexamic acid
