# Supplementary material for: Bleeding complications during cardiac electronic device implantation in patients receiving antithrombotic therapy: is there any value of local tranexamic acid?
Source: BMC Cardiovasc Disord. 2016 Apr 22;16:73. doi: 10.1186/s12872-016-0251-1 (PMC4841978; doi:10.1186/s12872-016-0251-1)
Supplement: Additional file 1: Table S1. — Characteristics of patients with pocket hematoma and/or major bleeding complications. Table S2. Baseline characteristics according to MBC. Table S3. Procedure related characteristics according to MBC. Table S4. Univariate and multivariate predictors of MBC. (DOCX 28 kb) [file 12872_2016_251_MOESM1_ESM.docx]

| **Table S1.** Characteristics of patients with pocket hematoma and/or major bleeding complications. | | | | | | | | | |
| --- | --- | --- | --- | --- | --- | --- | --- | --- | --- |
| **Patient** | **Age**  **(years)** | **Gender** | **Procedure** | **Topical TXA use** | **Pocket location** | **Pre-procedural antithrombotic therapy** | **INR** | **Complications** | **Interventions** |
| 1 | 78 | Male | NI of Dual-ICD | No | SQ | DAPT | 1.3 | HT+PE+Exitus | PC+Chest tube |
| 2 | 63 | Female | GEPR of CRT-D | No | SQ | W | 2.2 | PH | REOP |
| 3 | 72 | Male | NI of CRT-D | No | SQ | DAPT | 1.2 | PH | RBC-T |
| 4 | 76 | Male | NI of CRT-D | No | SQ | W plus DAPT | 2.0 | PH | RBC-T |
| 5 | 48 | Female | NI of CRT-D | No | SQ | W plus DAPT | 2.0 | PH | RBC-T |
| 6 | 65 | Male | GEPR of CRT-D | No | SQ | DAPT | 1.1 | PH | RBC-T |
| 7 | 65 | Female | NI of Dual-ICD | No | SQ | W plus DAPT | 2.2 | PH | RBC-T+REOP |
| 8 | 74 | Male | NI of CRT-D | No | SQ | DAPT | 0.9 | PH | RBC-T |
| 9 | 55 | Female | ULR of CRT-D | No | SQ | W plus DAPT | 2.1 | PH | RBC-T |
| 10 | 49 | Male | ULR of CRT-D | No | SQ | W | 2.5 | PH | RBC-T+REOP |
| 11 | 78 | Female | ULR of CRT-D | No | SQ | W plus DAPT | 2.1 | PH | RBC-T+REOP |
| 12 | 49 | Male | NI of CRT-D | No | SQ | DAPT | 1.0 | PE | PC |
| 13 | 66 | Male | NI of Dual-ICD | No | SQ | W plus DAPT | 2.0 | PH | RBC-T+REOP |
| 14 | 59 | Male | NI of CRT-D | No | SQ | W plus DAPT | 2.1 | PH | RBC-T+REOP |
| 15 | 80 | Male | NI of CRT-D | No | SQ | DAPT | 1.2 | PH | RBC-T |
| 16 | 49 | Male | NI of CRT-D | No | SQ | W | 2.0 | PH | RBC-T+REOP |
| 17 | 83 | Female | NI of CRT-D | No | SQ | W plus DAPT | 2.1 | PH | REOP |
| 18 | 64 | Male | NI of CRT-D | No | SQ | W plus DAPT | 2.4 | PH | None |
| 19 | 71 | Female | ULR of CRT-D | No | SQ | W plus DAPT | 2.1 | PH | None |
| 20 | 75 | Female | NI of CRT-D | No | SQ | DAPT | 1.2 | PH | None |
| 21 | 61 | Male | NI of Dual-ICD | No | SQ | W plus DAPT | 2.1 | PH | None |
| 22 | 58 | Female | NI of CRT-D | No | SQ | W plus DAPT | 2.0 | PH | None |
| 23 | 77 | Female | NI of CRT-D | No | SQ | DAPT | 1.1 | PH | None |
| 24 | 46 | Male | NI of CRT-D | No | SQ | DAPT | 1.0 | PH | None |
| 25 | 72 | Female | GEPR of CRT-D | Yes | SQ | W | 2.5 | PH | RBC-T |
| 26 | 53 | Female | NI of Dual-ICD | Yes | SQ | W plus DAPT | 2.4 | PH | RBC-T |
| 27 | 47 | Male | ULR of Dual-PM | Yes | SM | W plus DAPT | 2.2 | PH+PE | PC |
| 28 | 61 | Male | NI of Dual-ICD | Yes | SQ | W plus DAPT | 2.1 | PH | None |

CRT-D = cardiac resynchronization therapy-defibrillator; DAPT = dual antiplatelet therapy; Dual-ICD = dual chamber implantable cardioverter defibrillator; Dual-PM = dual chamber pacemaker; GEPR = generator exchange and/or pocket revision; HT = hemothorax; ICD = implantable cardioverter defibrillator; INR = international normalized ratio; NI = new implantation; PC = pericardiocentesis; PE = pericardial effusion; REOP = reoperation (pocket evacuation or revision); PH = pocket hematoma; RBC-T= red blood cells transfusion SM = submuscular; SQ = subcutaneous; ULR = upgrade and/or lead revision; W = warfarin.

| Table S2. Baseline characteristics according to MBC. | | | |
| --- | --- | --- | --- |
| Variables | **MBC positive group**  **(n = 20)** | **MBC negative group**  **(n = 115)** | **P-value** |
| Age | 64 ± 13 | 60 ± 11 | 0.118 |
| Male, n (%) | 12 (60.0) | 69 (60.0) | 1.0 |
| Body mass index (kg/m^2^) | 25.9 ± 6.2 | 25.3 ± 4.3 | 0.594 |
| Smoking, n (%) | 1 (5.0) | 21 (18.3) | 0.196 |
| Hypertension, n (%) | 15 (75.0) | 59 (51.3) | 0.085 |
| Diabetes mellitus, n (%) | 5 (25.0) | 41 (35.7) | 0.502 |
| Previous CABG, n (%) | 6 (30.0) | 37 (32.2) | 1.0 |
| Ejection fraction, % | 32.5 (25-39) | 34.0 (20-55) | 0.210 |
| COPD, n (%) | 2 (10.0) | 17 (14.8) | 0.738 |
| Hemoglobin, g/dL | 12.9 (10.8-15.1) | 12.8 (9.5-14.5) | 0.946 |
| Platelet count, K/mm^3^ | 246 ± 55 | 237 ±70 | 0.509 |
| BUN, mg/dL | 29.6 (25-45) | 31.6 (22-43) | 0.975 |
| Creatinine, mg/dL | 1.0 ± 0.4 | 1.1 ± 0.4 | 0.514 |
| Atrial fibrillation, n (%) | 8 (40.0) | 35 (30.4) | 0.557 |
| Metallic prosthetic valve, n (%) | 9 (45.0) | 45 (39.1) | 0.805 |
| LV thrombus, n (%) | 3 (15.0) | 6 (5.2) | 0.130 |
| Recent stent implantation, n (%) | 16 (80.0) | 47 (40.9) | 0.003 |
| *Medications* |  |  |  |
| ACEI/ARB, n (%) | 17 (85.0) | 78 (67.8) | 0.184 |
| Beta blocker, n (%) | 19 (95.0) | 93 (80.9) | 0.196 |
| Diuretic, n (%) | 17 (85.0) | 82 (71.3) | 0.315 |
| Spironolactone, n (%) | 14 (70.0) | 53 (46.1) | 0.083 |
| Statin, n (%) | 9 (45.0) | 53 (46.1) | 1.0 |
| Warfarin, n (%) | 4 (20.0) | 68 (59.1) | 0.003 |
| DAPT, n (%) | 6 (30.0) | 31 (27.0) | 0.992 |
| Warfarin plus DAPT, n (%) | 10 (50.0) | 16 (13.9) | 0.001 |

ACEI = angiotensin converting enzyme inhibitors; ARB = angiotensin receptor blockers; BUN = blood urea nitrogen; CABG = coronary artery bypass graft; COPD = chronic obstructive pulmonary disease; DAPT = dual antiplatelet therapy; LV = left ventricular; MBC = major bleeding complications.

| Table S3. Procedure related characteristics according to MBC. | | | |
| --- | --- | --- | --- |
| Characteristics | **MBC positive group**  **(n = 20)** | **MBC negative group**  **(n = 115)** | **P-value** |
| INR at the day of implant* | 2.1(2.0-2.2) | 2.1(1.9-2.5) | 0.778 |
| Generator exchange and/or pocket revision, n (%) | 3 (15.0) | 11 (9.6) | 0.436 |
| New implantation, n (%) | 14 (70.0) | 87 (75.7) | 0.796 |
| Upgrade and/or lead revision, n (%) | 3 (15.0) | 17 (14.8) | 1.0 |
| Pacemaker, n (%) | 1 (5.0) | 23 (20.0) | 0.108 |
| ICD, n (%) | 19 (95.0) | 92 (80.0) |  |
| *Number of leads implanted* |  |  |  |
| One, n (%) | 2 (10.0) | 24 (20.9) | 0.363 |
| Two, n (%) | 6 (30.0) | 44 (38.3) | 0.618 |
| Three, n (%) | 9 (45.0) | 36 (31.3) | 0.346 |
| Submuscular pocket, n (%) | 1 (5.0) | 4 (3.5) | 0.557 |
| *Venous route other than subclavian* |  |  |  |
| Axillary, n (%) | 2 (10.0) | 10 (8.7) | 0.692 |
| Cephalic, n (%) | 1 (5.0) | 6 (5.2) | 1.0 |
| Local tranexamic acid use | 3 (15.0) | 49 (42.6) | 0.036 |

DAPT = dual antiplatelet therapy; ICD = implantable cardioverter defibrillator; INR = international normalized ratio; MBC = major bleeding complications. * The median INR level of patients with warfarin continuation strategy.

| Table S4. Univariate and multivariate predictors of MBC. | | | | | | |
| --- | --- | --- | --- | --- | --- | --- |
|  | **Univariate** | | | **Multivariate** | | |
| Variables | **OR** | **95%CI** | **P-value** | **OR** | **95%CI** | **P-value** |
| Hypertension | 2.847 | 0.971-8.353 | 0.057 |  |  |  |
| History of recent stent implantation | 5.787 | 1.820-18.406 | 0.003 |  |  |  |
| Spironolactone use | 2.730 | 0.980-7.602 | 0.055 |  |  |  |
| Periprocedural  warfarin use | 0.173 | 0.054-0.550 | 0.003 |  |  |  |
| Periprocedural warfarin plus DAPT use | 6.187 | 2.224-17.216 | <0.001 | 8.144 | 2.589-25.618 | <0.001 |
| ICD device | 0.211 | 0.027-1.655 | 0.139 |  |  |  |
| Topical TXA use during CIED implantation | 0.238 | 0.066-0.856 | 0.028 | 0.170 | 0.042-0.690 | 0.013 |

CI = confidence interval; CIED = cardiac electronic device implantation; DAPT = dual antiplatelet therapy; ICD = implantable cardioverter defibrillator; MBC = major bleeding complications; TXA = tranexamic acid.
